# Evaluation of parent-child relationships using the flannel-graph in children with autism spectrum disorders

**DOI:** 10.1192/j.eurpsy.2021.593

**Published:** 2021-08-13

**Authors:** A. Koval-Zaytsev, E. Furaeva

**Affiliations:** 1 Department Of Child Psychiatry, Federal State Budgetary Scientific Institution “Mental Health Research Center”, Moscow, Russian Federation; 2 Department Of Child Clinical Psychology, Moscow State School №2200, Moscow, Russian Federation

**Keywords:** autism spectrum disorders, child-parent relationships, the dynamics of development

## Abstract

**Introduction:**

The study of child-parent relationships in families raising children with autism spectrum disorders (ASD) and the assessment of the dynamics of development of a child with ASD are necessary to provide timely psychological personalized assistance to such families.

**Objectives:**

To study child-parent relations based on the mother’s behavior in an experimental play settings, in families raising children with ASD.

**Methods:**

The child-parent relationship was being analyzed through the flannel graph that the child had previously created with fairy tale characters. This evaluation assessed child’s independence level, parent and child engagement levels. The following surveys were used to support these theses: “The interaction of the parents and the child” (IPC) and “Child Rejection Scale” (CRS). The survey sample size included 104 mothers and their children, half were with ASD (average age 7.1), and half were typically developed children (average age 7).

**Results:**

Typically developing children mastered the flannel graph exercise assessment independently; 30% children with ASD did not complete the exercise due to the severity of the disease. Subsequently, among the mothers whose children have ASD 26% refused to participate and the facilitator helped them instead. Statistically significant differences were found on the IPC scales – satisfaction with family relationships; emotional proximity; disciplinary confrontation in the family. CRS identified 13% of mothers of children with ASD with pronounced rejection of children.

**Conclusions:**

The study of child-parent relationships using flannel graph assessment can be helpful in a comprehensive research of ASD and in programming a psychocorrectional work with children, using the child’s proximal development.

